# Using Games to Promote English as a Foreign Language Learners’ Willingness to Communicate: Potential Effects and Teachers’ Attitude in Focus

**DOI:** 10.3389/fpsyg.2021.762447

**Published:** 2021-10-12

**Authors:** Fei Liu, Balachandran Vadivel, Ehsan Rezvani, Ehsan Namaziandost

**Affiliations:** ^1^School of Foreign Studies, Northwestern Polytechnical University, Xi’an, China; ^2^Department of English, Cihan University-Duhok, Kurdistan Region, Iraq; ^3^English Department, Islamic Azad University of Isfahan (Khorasgan Branch), Isfahan, Iran; ^4^Department of English, Islamic Azad University of Shahrekord, Shahrekord, Iran

**Keywords:** games, willingness to communicate (WTC), EFL learners, teachers’ attitude, motivation

## Abstract

This study aims to find out the role of games in promoting students’ willingness to communicate (WTC) and their teachers’ attitude toward it. In order to collect the data, the researchers employed a 28-item questionnaire which was given to 60 English as a Foreign Language (EFL) learners in an English institute. Then, the students were randomly divided into two groups of 30 learners functioning as control and experimental groups. The students in the experimental group received games in their language lessons and classes, while control group learners did not. At the end of the term, the same questionnaire was given to the students to know if playing games had a significant impact on their WTC. In addition, the teachers were asked to answer a 30-item questionnaire to investigate their attitudes toward playing games in language classes. The results showed that most of the teachers in this study believe that games have a positive influence on the students’ attitudes towards learning English and that using them in class serves many educational purposes. In addition, games played a significant role in improving the EFL leaners’ willingness to communicate. In the light of these findings, the researchers suggested using games as energizers and practical activities at the end of class not only to improve enthusiasm for learning, but also to improve the learners’ WTC.

## Introduction

Lack of inspiration and motivation, using traditional methods of learning and teaching, being discouraged by their teachers and instructors, and having a fear of not learning English easily are some of the fundamental difficulties in learning to speak English correctly and fluently. Due to not having enough self-confidence, learners - especially young ones - face more problems in this area. To overcome this trouble, some teachers believe that games, specifically integrating ones into the teaching process, could increase students’ desire and willingness to learn more and communicate in classrooms in all levels and from different interests.

As a matter of fact, learners want active, fun, and interesting enough activities to get motivated to learn more. Research in this subject has signified various advantages of integrating games into language teaching: games emphasize the meaning in language learning, thus, learners will better remember the language they learnt (Tuan and Doan, 2010); games enable children to develop physically, socially, emotionally, and cognitively as well as being enjoyable and fun, either as a competition or cooperation with clearly defined goals and rules (Read, 2007); and games provide a fun and comfortable environment in which learners are more motivated to take risks in language practice (Wright et al., 2006).

This study was done in order to find out whether playing educational games based on the learners’ levels can play a significant role in encouraging them to communicate instead of using old, boring, and traditional methods in English classrooms. This study aims to understand whether games can trigger students’ willingness to learn by playing games in class and try to relieve associated learning problems. The basic function of games is to intensify human experiences in ways that are relatively safe. The theory of games might be called the mathematics of competition and cooperation. Situations are analyzed in terms of gains and losses of opposing players. They are applied in various aspects of life and different areas of study such as economics, mathematics, science, and language. McFarlane et al. (2002) agreed with Sim City (2002) in the respect that they all showed the importance of cooperative games, competitive games, and communication games as one of the most important ways to teach efficiently in a language class.

This study aims to find out the effectiveness of using games in teaching English and their role in promoting students’ attitudes towards learning English. In addition, it aims to discover how useful and practical educational games can be in promoting EFL learners to communicate in class and break the ice from the teachers’ perspective. Moreover, it aims to know teachers’ attitudes in this case.

### Research Questions

RQ1: Does using games have a significant effect on Iranian EFL learners’ WTC?
RQ2: What are Iranian EFL teachers’ attitude in regard to using games in increasing students’ WTC?

## Litreture Review

A lot of research has been carried out on the method of teaching using games and other types of icebreakers; by reviewing the educational related literature, the researchers were able to come up with a number of studies which strongly supported the use of games, as they are considered a welcome break from the usual and boring routine of a language class.

Quinn (2011) studied the fact that serious games create a hands-on, minds-on opportunity that allow players to actively focus on, create, and change a scenario whilst simultaneously learning about consequences of choice in the situation. When students become more engaged and committed to succeeding in the game, they become more willing to learn about the scenario the situation is taking place in.

Schuna (2010) proved that playing educational games also helps learners with focus, self-esteem, and memory. Educational games can help children focus because they are being patient while waiting to advance to the next level. Playing these games helps their self- esteem because sometimes they get a quicker reaction from the game system, and they can really see how they have accomplished something.

Johnson (2005) supported McFarlane and Sakellariou (2002) who see game play as inherently valuable, leading to the development of a range of skills and competencies that may be transferred to other social and work-related uses of digital technologies. Macedonia (2005) argued whether learning a foreign language is declarative or procedural. She concluded that the process of learning FL is procedural. One of the methods used is language games which are employed in a targeted way to proceduralise foreign language. Moreover, they bring a sense of fun and a positive attitude towards learning and facilitate the learning process. Positive emotions promote learning not only in our perception but also from a neurological perspective. In contrast, negative emotions restrain information flow.

Kamra (2010) concluded that using games is an efficient way to teach English in the classroom. Following this method, you get the best results in the classroom. It increases students’ motivation. Games prepare young learners for life, and they acquire positive social attitudes. Games teach sharing, helping each other, and working as a team. A child learns by doing, living, trying, and imitating. So this kind of learning is lasting. During games, some feelings, such as the pleasure of winning and the fear of losing, may arise. This gives the teacher an idea about the student’s character. So, games are must-have activities for hardworking teachers. This is in line with Buckingham (2003).

In conclusion, Prensky argued that children are naturally motivated to play games. Serious games are interactive play that teach students goals, rules, adaptation, problem solving, and interaction, all represented as a story. They allow them to learn by providing enjoyment, passionate involvement, structure, motivation, ego gratification, adrenaline, creativity, social interaction, and emotion. “Playing has a deep biological, evolutionarily important, function, which has to be done specifically with learning.”(Prensky, 2011; P. 40).

Having reviewed the relevant research projects and mentioned their results and findings, we now turn to the present study’s purpose and research questions. The purpose of this study is therefore to use games to promote EFL learners’ willingness to communicate in an Iranian context. The study focuses on the roles that games play in children’s classrooms to know if playing educational games can lead the students to be more active and motivated to communicate more in class.

## Methodology

### Participants

The participants were one hundred students chosen from intermediate classes of the English institutes. They were both male and female students between 18 and 35 years old. In order to have homogenous participants, OPT was run and 60 intermediate students were chosen. The other participants of the study were six English teachers (three females and three males) teaching in that institution. All these teachers had teaching experience with young students of at least 10 years. They all had either a B.A or M.A. from ELT departments of different universities in Iran. Their majors were English teaching and English Literature. All the teachers were taught how to apply these games and when to play them.

### Instruments

In carrying out this research, four different instruments were applied. They were Oxford Placement Test (OPT), Willingness to Communicate (WTC) pre and post-test, attitude questionnaire, and finally ten educational games. The first three instruments were applied in all classes, but games were only used in the experimental group.

### Oxford Placement Test

The OPT (Allan, 2005) consists of 200 items including 100 grammar items. For the purpose of this study, only the grammar part was used, and so through a pilot study, its reliability was estimated. The Kr-21 reliability formula showed a reliability of 0.78, which is a rather acceptable reliability for using the test. It took about 55 minutes for students to complete the test. After administrating the test, the obtained results were estimated based on the OPT-associated rating levels chart and those who received 70 or more in this test were considered as intermediate learners.

### Willingness to Communicate Pre-test and Post-test

In order to collect the required data, all of the participants were asked to answer a WTC questionnaire which had had five parts; scores between 0 and 4 were given to them: Never (0), Rarely (1), Sometimes (2), Often (3), and Almost always (4) as the pre-test. After carrying out the treatment the participants were asked to answer the same questionnaire. The questionnaire was obtained from Gol et al. (2014).

### Attitude Questionnaire

The present study is concerned with investigating teachers’ attitude toward playing games in the classrooms. Therefore, all six teachers were asked to check their ideas. The questionnaire had 30 questions with Likert Scale of five. The questionnaire had five parts and scores between 0 and 5 were given to them: Very low (1), Low (2), Moderate (3), High (4), and Very high (5). The questionnaire was obtained from Mahmoud and Tanni (2012).

### Educational Games

Ten educational games were selected by the researchers to be played in the experimental group in order to investigate their role on learners’ willingness to communicate.

### Procedures

In the study, a questionnaire with a five- point Likert-type rating scale was formed by the researchers in order to investigate what practicing teachers think about WTC through games. The questionnaire was administered in their classrooms. The internal consistency reliability of the learners’ questionnaire was checked based on the Cronbach’s alpha. On the other hand, the students were asked to answer two (pre and post) tests to know if the games were useful.

One hundred English learners were chosen to participate in this study. Their level of proficiency was intermediate since they all passed the same levels in that institute. To be sure about it, OPT was run to have a homogenous group based on the scores they got and eventually sixty of the students were chosen as participants of the study. Then WTC pre-test was performed on all the students to know their willingness to communicate (WTC) during class time.

Afterward, the students were divided into two groups, each containing thirty students. The group which underwent the treatment was called game group and the other thirty students were called control group. Then, the educational games were played in the game group at the end of each class, but no games were applied in the control group, to investigate whether games encouraged learners’ willingness to speak English as well as improve their speaking. After that, all the students were asked to check their WTC in the same questionnaire as the post-test of the study. Finally, in order to investigate the teachers’ attitude toward playing games, they were asked to answer the questionnaire.

### Data Analysis and Results

A willingness to communicate questionnaire was given to the control group and experimental group to investigate their willingness to communicate before doing any treatment. The questionnaire had twenty-eight questions. The questionnaire had five parts and scores of 0 to 4 were given to them: Never with the score of zero, Rarely with the score of one, Sometimes with the score of two, Often with the score of three, and Almost always with the score of four.

Table 1 shows the frequency and the percentage of responses to the questionnaire for students in the control group in the pre-test phase. Average scores earned by learners for each question are also displayed in all the tables.

**TABLE 1:** Distribution of the responses of the control group to the questionnaire before Treatment.

	**Never**		**Rarely**		**Sometimes**		**Often**		**Almost always**		**Mean**	**Std. deviation**
	**Frequency**	**Percentage**	**frequency**	**Percentage**	**Frequency**	**Percentage**	**Frequency**	**percentage**	**Frequency**	**Percentage**		
*Q1*	5	16.7	7	23.3	9	30.0	7	23.3	2	6.7	1.8	1.2
*Q2*	3	10.0	4	13.3	18	60.0	4	13.3	1	3.3	1.9	0.9
*Q3*	0	0.0	10	33.3	11	36.7	7	23.3	2	6.7	2.0	0.9
*Q4*	1	3.3	7	23.3	13	43.3	7	23.3	2	6.7	2.1	0.9
*Q5*	2	6.7	9	30.0	12	40.0	7	23.3	0	0.0	1.8	0.9
*Q6*	1	3.3	8	26.7	14	46.7	4	13.3	3	10.0	2.0	1.0
*Q7*	3	10.0	5	16.7	14	46.7	6	20.0	2	6.7	2.0	1.0
*Q8*	3	10.0	11	36.7	9	30.0	5	16.7	2	6.7	1.7	1.1
*Q9*	0	0.0	9	30.0	11	36.7	10	33.3	0	0.0	2.0	0.8
*Q10*	3	10.0	8	26.7	13	43.3	5	16.7	1	3.3	1.8	1.0
*Q11*	1	3.3	11	36.7	11	36.7	6	20.0	1	3.3	1.8	0.9
*Q12*	3	10.0	4	13.3	13	43.3	8	26.7	2	6.7	2.1	1.0
*Q13*	0	0.0	10	33.3	15	50.0	5	16.7	0	0.0	1.8	0.7
*Q14*	3	10.0	9	30.0	8	26.7	8	26.7	2	6.7	1.9	1.1
*Q15*	1	3.3	8	26.7	13	43.3	4	13.3	4	13.3	2.1	1.0
*Q16*	1	3.3	6	20.0	14	46.7	9	30.0	0	0.0	2.0	0.8
*Q17*	2	6.7	3	10.0	15	50.0	8	26.7	2	6.7	2.2	0.9
*Q18*	2	6.7	8	26.7	13	43.3	5	16.7	2	6.7	1.9	1.0
*Q19*	0	0.0	9	30.0	13	43.3	7	23.3	1	3.3	2.0	0.8
*Q20*	3	10.0	5	16.7	13	43.3	8	26.7	1	3.3	2.0	1.0
*Q21*	3	10.0	8	26.7	10	33.3	8	26.7	1	3.3	1.9	1.0
*Q22*	0	0.0	6	20.0	11	36.7	11	36.7	2	6.7	2.3	0.9
*Q23*	4	13.3	7	23.3	10	33.3	9	30.0	0	0.0	1.8	1.0
*Q24*	2	6.7	5	16.7	12	40.0	10	33.3	1	3.3	2.1	1.0
*Q25*	1	3.3	9	30.0	10	33.3	9	30.0	1	3.3	2.0	0.9
*Q26*	7	23.3	7	23.3	8	26.7	8	26.7	0	0.0	1.6	1.1
*Q27*	2	6.7	4	13.3	15	50.0	7	23.3	2	6.7	2.1	1.0
*Q28*	1	3.3	14	46.7	11	36.7	3	10.0	1	3.3	1.6	0.9

Table 1 revealed that the highest mean was related to question 22 (“I ask the teacher a question in class.”) which was 2.3 and the lowest mean belonged to questions 26 and 28 which was 1.6. (26. “I participate in pair activities in class.”, 28. “I chat with my classmates out of class.”). The 23rd question shows that students were most eager to talk in that situation. On the other hand, the 26th and 28th questions demonstrated that they were not willing to communicate in these situations.

Table 2 displayed that the highest mean was related to question 22 (“I ask the teacher a question in class.”) which was 2.4 and the lowest mean was 1.5 and belonged to question 18 (“I dislike some of my classmates.”). Therefore, asking the teacher a question was mostly used and their dislikes of their classmates was used the least. Table 3 shows the analysis of data on the WTC post-test.

**TABLE 2:** Distribution of the responses of the experimental group to the questionnaire before treatment.

	**Never**		**Rarely**		**Sometimes**		**Often**		**Almost always**		**Mean**	**Std. deviation**
	**Frequency**	**Percentage**	**Frequency**	**Percentage**	**Frequency**	**Percentage**	**Frequency**	**percentage**	**Frequency**	**Percentage**		
*Q1*	2	6.7	9	30.0	10	33.3	6	20.0	3	10.0	2.0	1.1
*Q2*	2	6.7	9	30.0	6	20.0	11	36.7	2	6.7	2.1	1.1
*Q3*	1	3.3	5	16.7	13	43.3	10	33.3	1	3.3	2.2	0.9
*Q4*	0	0.0	11	36.7	14	46.7	3	10.0	2	6.7	1.9	0.9
*Q5*	1	3.3	4	13.3	18	60.0	7	23.3	0	0.0	2.0	0.7
*Q6*	1	3.3	10	33.3	12	40.0	6	20.0	1	3.3	1.9	0.9
*Q7*	3	10.0	7	23.3	12	40.0	6	20.0	2	6.7	1.9	1.1
*Q8*	0	0.0	12	40.0	11	36.7	6	20.0	1	3.3	1.9	0.9
*Q9*	1	3.3	9	30.0	13	43.3	7	23.3	0	0.0	1.9	0.8
*Q10*	2	6.7	8	26.7	13	43.3	5	16.7	2	6.7	1.9	1.0
*Q11*	2	6.7	7	23.3	11	36.7	9	30.0	1	3.3	2.0	1.0
*Q12*	0	0.0	6	20.0	16	53.3	7	23.3	1	3.3	2.1	0.8
*Q13*	2	6.7	7	23.3	11	36.7	8	26.7	2	6.7	2.0	1.0
*Q14*	2	6.7	8	26.7	10	33.3	8	26.7	2	6.7	2.0	1.1
*Q15*	3	10.0	5	16.7	10	33.3	12	40.0	0	0.0	2.0	1.0
*Q16*	0	0.0	10	33.3	10	33.3	8	26.7	2	6.7	2.1	0.9
*Q17*	1	3.3	12	40.0	8	26.7	8	26.7	1	3.3	1.9	1.0
*Q18*	6	20.0	7	23.3	13	43.3	4	13.3	0	0.0	1.5	1.0
*Q19*	2	6.7	8	26.7	11	36.7	7	23.3	2	6.7	2.0	1.0
*Q20*	2	6.7	11	36.7	8	26.7	7	23.3	2	6.7	1.9	1.1
*Q21*	2	6.7	10	33.3	12	40.0	4	13.3	2	6.7	1.8	1.0
*Q22*	0	0.0	3	10.0	14	46.7	11	36.7	2	6.7	2.4	0.8
*Q23*	4	13.3	9	30.0	11	36.7	4	13.3	2	6.7	1.7	1.1
*Q24*	1	3.3	8	26.7	15	50.0	4	13.3	2	6.7	1.9	0.9
*Q25*	1	3.3	9	30.0	11	36.7	9	30.0	0	0.0	1.9	0.9
*Q26*	3	10.0	7	23.3	10	33.3	6	20.0	3	10.0	2.1	1.3
*Q27*	4	13.3	8	26.7	10	33.3	8	26.7	0	0.0	1.7	1.0
*Q28*	1	3.3	7	23.3	7	23.3	14	46.7	1	3.3	2.2	1.0

**TABLE 3:** Distribution of the responses of the control group to the questionnaire after treatment.

	**Never**		**Rarely**		**Sometimes**		**Often**		**Almost always**		**Mean**	**Std. deviation**
	**Frequency**	**Percentage**	**Frequency**	**Percentage**	**Frequency**	**Percentage**	**Frequency**	**Percentage**	**Frequency**	**Percentage**		
*Q1*	5	16.7	7	23.3	10	33.3	7	23.3	1	3.3	1.7	1.1
*Q2*	2	6.7	5	16.7	18	60.0	3	10.0	2	6.7	1.9	0.9
*Q3*	0	0.0	8	26.7	13	43.3	7	23.3	2	6.7	2.1	0.9
*Q4*	1	3.3	6	20.0	13	43.3	8	26.7	2	6.7	2.1	0.9
*Q5*	1	3.3	10	33.3	12	40.0	7	23.3	0	0.0	1.8	0.8
*Q6*	0	0.0	9	30.0	14	46.7	4	13.3	3	10.0	2.0	0.9
*Q7*	1	3.3	6	20.0	15	50.0	6	20.0	2	6.7	2.1	0.9
*Q8*	2	6.7	13	43.3	9	30.0	4	13.3	2	6.7	1.7	1.0
*Q9*	1	3.3	9	30.0	12	40.0	8	26.7	0	0.0	1.9	0.8
*Q10*	4	13.3	9	30.0	12	40.0	4	13.3	1	3.3	1.6	1.0
*Q11*	1	3.3	9	30.0	13	43.3	6	20.0	1	3.3	1.9	0.9
*Q12*	3	10.0	4	13.3	14	46.7	8	26.7	1	3.3	2.0	1.0
*Q13*	0	0.0	11	36.7	14	46.7	5	16.7	0	0.0	1.8	0.7
*Q14*	2	6.7	10	33.3	9	30.0	8	26.7	1	3.3	1.9	1.0
*Q15*	0	0.0	8	26.7	14	46.7	4	13.3	4	13.3	2.1	1.0
*Q16*	1	3.3	6	20.0	14	46.7	9	30.0	0	0.0	2.0	0.8
*Q17*	2	6.7	3	10.0	15	50.0	8	26.7	2	6.7	2.2	0.9
*Q18*	2	6.7	6	20.0	15	50.0	5	16.7	2	6.7	2.0	1.0
*Q19*	0	0.0	9	30.0	12	40.0	8	26.7	1	3.3	2.0	0.9
*Q20*	2	6.7	6	20.0	13	43.3	8	26.7	1	3.3	2.0	0.9
*Q21*	2	6.7	8	26.7	11	36.7	8	26.7	1	3.3	1.9	1.0
*Q22*	0	0.0	5	16.7	12	40.0	11	36.7	2	6.7	2.3	0.8
*Q23*	4	13.3	7	23.3	10	33.3	9	30.0	0	0.0	1.8	1.0
*Q24*	2	6.7	4	13.3	14	46.7	9	30.0	1	3.3	2.1	0.9
*Q25*	2	6.7	7	23.3	11	36.7	9	30.0	1	3.3	2.0	1.0
*Q26*	5	16.7	11	36.7	8	26.7	6	20.0	0	0.0	2.1	0.9
*Q27*	1	3.3	5	16.7	15	50.0	7	23.3	2	6.7	1.5	1.0
*Q28*	1	3.3	15	50.0	10	33.3	3	10.0	1	3.3	1.6	0.9

Table 2 revealed that the highest mean was related to question 22 (“I ask the teacher a question in class.”) which was 2.3. As it can be seen, this mean was the same as the mean of this question in the pre-test. The lowest mean belonged to the 27th question (“I help others answer a question.”) which was 1.5. Therefore, the results showed that learners’ question of the teacher was most used and helping classmates in answering was the least used among the learners in the control group.

Table 4 demonstrated that the highest mean was related to question number 3 (“the topic is interesting”) which was 2.4. and the lowest score was related to question 9 (“an assignment is being discussed.”) which was 1.9. Therefore, being interested in a topic was most checked by the learners and discussing the assignments was used the least by the learners in the experimental group.

**TABLE 4:** Distribution of the responses of the experimental group to the questionnaire after treatment.

	**Never**		**Rarely**		**Sometimes**		**Often**		**Almost always**		**Mean**	**Std. deviation**
	**Frequency**	**Percentage**	**Frequency**	**Percentage**	**Frequency**	**Percentage**	**Frequency**	**Percentage**	**Frequency**	**Percentage**		
*Q1*	0	0.0	8	26.7	12	40.0	7	23.3	3	10.0	2.2	0.9
*Q2*	0	0.0	11	36.7	6	20.0	11	36.7	2	6.7	2.1	1.0
*Q3*	0	0.0	3	10.0	14	46.7	11	36.7	2	6.7	2.4	0.8
*Q4*	0	0.0	11	36.7	14	46.7	3	10.0	2	6.7	1.9	0.9
*Q5*	0	0.0	5	16.7	18	60.0	7	23.3	0	0.0	2.1	0.6
*Q6*	0	0.0	6	20.0	15	50.0	8	26.7	1	3.3	2.1	0.8
*Q7*	0	0.0	7	23.3	13	43.3	7	23.3	3	10.0	2.2	0.9
*Q8*	0	0.0	12	40.0	11	36.7	6	20.0	1	3.3	2.0	0.9
*Q9*	0	0.0	10	33.3	12	40.0	7	23.3	1	3.3	1.9	0.9
*Q10*	0	0.0	10	33.3	13	43.3	5	16.7	2	6.7	2.0	0.9
*Q11*	0	0.0	9	30.0	11	36.7	8	26.7	2	6.7	2.1	0.9
*Q12*	0	0.0	5	16.7	17	56.7	4	13.3	4	13.3	2.2	0.9
*Q13*	0	0.0	9	30.0	11	36.7	5	16.7	5	16.7	2.2	1.1
*Q14*	0	0.0	9	30.0	11	36.7	4	13.3	6	20.0	2.2	1.1
*Q15*	0	0.0	8	26.7	10	33.3	9	30.0	3	10.0	2.2	1.0
*Q16*	0	0.0	6	20.0	12	40.0	9	30.0	3	10.0	2.3	0.9
*Q17*	0	0.0	8	26.7	10	33.3	8	26.7	4	13.3	2.3	1.0
*Q18*	0	0.0	9	30.0	12	40.0	5	16.7	4	13.3	2.1	1.0
*Q19*	0	0.0	10	33.3	11	36.7	4	13.3	5	16.7	2.1	1.1
*Q20*	0	0.0	11	36.7	7	23.3	6	20.0	6	20.0	2.2	1.2
*Q21*	0	0.0	11	36.7	13	43.3	2	6.7	4	13.3	2.0	1.0
*Q22*	0	0.0	6	20.0	13	43.3	8	26.7	3	10.0	2.3	0.9
*Q23*	0	0.0	7	23.3	15	50.0	6	20.0	2	6.7	2.1	0.8
*Q24*	0	0.0	8	26.7	15	50.0	4	13.3	3	10.0	2.1	0.9
*Q25*	0	0.0	10	33.3	11	36.7	9	30.0	0	0.0	2.0	0.8
*Q26*	0	0.0	9	30.0	11	36.7	7	23.3	3	10.0	2.1	1.0
*Q27*	0	0.0	6	20.0	13	43.3	11	36.7	0	0.0	2.2	0.7
*Q28*	0	0.0	8	26.7	7	23.3	14	46.7	1	3.3	2.3	0.9

Table 5 shows the mean and Std. Deviation of every question between the learners of the two groups in pre-test and post-test. The results of the table revealed that, in the experimental group, there was an increase in the mean score from the pre-test to the post-test. On the other hand, in the control group, the answers to questions 1 (“the class is engaged in an open discussion”), 9 (“an assignment is being discussed”), 10 (“I am comfortable with the subject matter”), 12 (“no one else is talking”), and 26 (“I participate in pair activities in class.”) experienced a great decrease. Their means were 1.7, 1.9, 1.6, 2, and 1.5 respectively. However, the answers to questions 3 (“the topic is interesting”), 7 (“everyone is talking”), 11 (“the topic is based on my experience”), and 18 (“I dislike some of my classmates”) enjoyed a great increase. Their means were 2.1, 2.1, 1.9, and 2.0 respectively. In other questions, the average score of the questions was not changed.

**TABLE 5:** Mean and std. deviation of the questionnaire scores for learners of the control and experimental groups before and after the treatment.

	**Control**				**Experimental**			
	**Pretest**		**Posttest**		**Pretest**		**Posttest**	
	**M**	**SD**	**M**	**SD**	**M**	**SD**	**M**	**SD**
*Q1*	1.8	1.2	1.7	1.1	2.0	1.1	2.2	0.9
*Q2*	1.9	0.9	1.9	0.9	2.1	1.1	2.1	1.0
*Q3*	2.0	0.9	2.1	0.9	2.2	0.9	2.4	0.8
*Q4*	2.1	0.9	2.1	0.9	1.9	0.9	1.9	0.9
*Q5*	1.8	0.9	1.8	0.8	2.0	0.7	2.1	0.6
*Q6*	2.0	1.0	2.0	0.9	1.9	0.9	2.1	0.8
*Q7*	2.0	1.0	2.1	0.9	1.9	1.1	2.2	0.9
*Q8*	1.7	1.1	1.7	1.0	1.9	0.9	1.9	0.9
*Q9*	2.0	0.8	1.9	0.8	1.9	0.8	2.0	0.9
*Q10*	1.8	1.0	1.6	1.0	1.9	1.0	2.0	0.9
*Q11*	1.8	0.9	1.9	0.9	2.0	1.0	2.1	0.9
*Q12*	2.1	1.0	2.0	1.0	2.1	0.8	2.2	0.9
*Q13*	1.8	0.7	1.8	0.7	2.0	1.0	2.2	1.1
*Q14*	1.9	1.1	1.9	1.0	2.0	1.1	2.2	1.1
*Q15*	2.1	1.0	2.1	1.0	2.0	1.0	2.2	1.0
*Q16*	2.0	0.8	2.0	0.8	2.1	0.9	2.3	0.9
*Q17*	2.2	0.9	2.2	0.9	1.9	1.0	2.3	1.0
*Q18*	1.9	1.0	2.0	1.0	1.5	1.0	2.1	1.0
*Q19*	2.0	0.8	2.0	0.9	2.0	1.0	2.1	1.1
*Q20*	2.0	1.0	2.0	0.9	1.9	1.1	2.2	1.2
*Q21*	1.9	1.0	1.9	1.0	1.8	1.0	2.0	1.0
*Q22*	2.3	0.9	2.3	0.8	2.4	0.8	2.3	0.9
*Q23*	1.8	1.0	1.8	1.0	1.7	1.1	2.1	0.8
*Q24*	2.1	1.0	2.1	0.9	1.9	0.9	2.1	0.9
*Q25*	2.0	0.9	2.0	1.0	1.9	0.9	2.0	0.8
*Q26*	1.6	1.1	1.5	1.0	2.1	1.3	2.1	1.0
*Q27*	2.1	1.0	2.1	0.9	1.7	1.0	2.2	0.7
*Q28*	1.6	0.9	1.6	0.9	2.2	1.0	2.3	0.9

In order to answer the first question some procedures were applied. The above-mentioned results revealed that using games has a significant effect on Iranian EFL learners’ willingness to communicate. Playing educational games in the experimental group has demonstrated that the educational games can really increase Iranian leaners’ willingness to communicate and motivate them to talk more in class and share their information and experiences more. On the other hand, not playing educational games in the control group and teaching those without any treatment revealed that their willingness to communicate did not really improve. Therefore, by playing games in EFL classes, leaners can get more enthusiastic to talk and have discussions in class.

Based on Table 6 the average score of the learners in the control group was 54.20 and their Std. Deviation was ± 4.11 in the pre-test. These scores were increased in the post-test; the mean increased to 54.37 and the Std. Deviation became ± 4.16. The means and Std. Deviation of pre-test and post-test do not show a great difference in the control group.

**TABLE 6:** Average score of learners in both groups in pre-test and post-test.

**WTC score**	**Pretest**		**Posttest**	
	**Mean**	**SD**	**Mean**	**SD**
Control	54.20	4.11	54.37	4.16
Experimental	54.73	5.60	59.77	5.15

On the other hand, as the table shows, the mean and Std. Deviation of the experimental group in pre-test were 54.73 and ± 5.60. The average of the experimental group was increased in the post-test to 59.77 and their Std. Deviation became ± 5.15.

**FIGURE 1:**
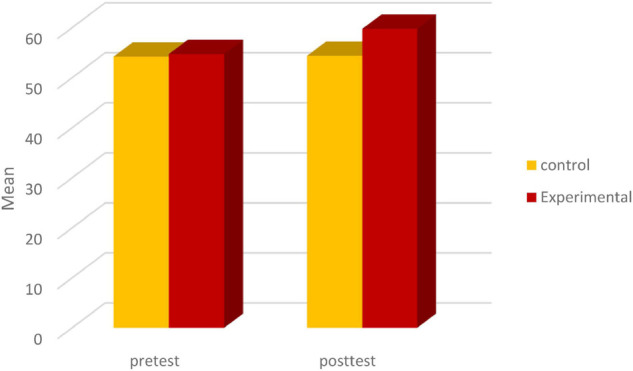
The mean of the scores of WTC between learners in pre-test and post-test.

Figure 1 shows the mean of the control and experimental group in pre-test and post-test.

In order to compare the mean of pre-test and post-test of the learners in the control group, a paired sample t-test was run. Table 7 shows the result of the comparison. Paired-sample t-test results showed no significant difference between mean scores of the learners in pre-test and post-test [t (29) = 0.556, *p* = 0.582]. The difference between pre-test and post-test was 0.167, which was not really different. It means that before and after the course their willingness to communicate did not change.

**TABLE 7:** Results of paired- sample t-test in comparison of mean score of learners in pre-test and post-test of the control group.

**Variable**	**Group**	**Mean difference (post-test-pre-test)**	**Std. deviation**	**T**	**Df**	***p*-value**
WTC score	Control	0.167	1.642	0.556	29	0.582

Another paired sample *t*-test was run (Table 8) to compare the scores of the experimental group’s pre-test and post-test. In the experimental group, the results of t-test showed a significant difference between the mean score of their pre-test and post-test [t (29) = 7.750, *p* < 0.001]. The mean of the post-test was significantly higher than the mean of the pre-test. This difference was about 5.033, which was a great difference. Therefore, the treatment had played a significant role in improving learner’s willingness to communicate and motivated them to talk more during class time.

**TABLE 8:** Results of paired- sample t-test in comparison of mean score of learners in pre-test and post-test of the experimental group.

**Variable**	**Group**	**Mean difference (post-test-pre-test)**	**Std. deviation**	**T**	**Df**	***p*-value**
WTC score	Control	5.033	3.557	7.750	29	<0.001

In order to compare the difference between pre-test and post-test scores of both groups, an independent t-test was run. As Table 9 shows, the average difference of the mean of the control group in pre-test and post-test was 0.17 and its Std. Deviation was 1.64. In other words, the average difference between the pre-test and post-test scores of the control group was increased by about 0.17, which was not really significant.

**TABLE 9:** Independent T-test results in comparison of difference between pre-test and post-test scores of the control group and the experimental group.

**Variable**	**Group**	***N***	**Mean**	**Std. deviation**	**T**	**Df**	***p*-value**
Reading ability (post-test-pre-test)	Control	30	0.17	1.64	−6.804	58	<0.001
	Experimental	30	5.03	3.56			

Table 9 shows that the average difference between two stages of pre-test and post-test in the experimental group was 5.03 and its Std. Deviation was 3.56. It indicates that learners’ scores in the experimental group had an average increase of 5.03. The average score differences between the two groups were compared by independent t-test. The results of an average of the differences between pre-test and post-test of the experimental group was significantly higher than the average of the control group [*t* (58) = −6.804, *p* < 0.001]. Therefore, the experimental group took the advantage of playing educational games in willingness to communicate.

### Teachers’ Attitude Results and Discussions

In order to collect the required data of the study, a questionnaire with 30 questions was given to the six teachers to investigate Iranian EFL teachers’ attitudes in regard to using games to increase students’ willingness to communicate. Then the collected data were analyzed, and Tables 10, 11 show this analysis. The questionnaire had five parts and scores between 0 and 5 were given to them: Very low with the score of one, Low with the score of two, Moderate with the score of three, High with the score of four, and Very high with the score of five. Table 10 shows the frequency and percentage of answers to different scales of the questionnaire.

**TABLE 10:** Frequency distribution of teachers’ answers to the questionnaire.

	**Very low**		**Low**		**Moderate**		**High**		**Very high**		**Mean**	**Std. deviation**	***p*-value**
	**Frequency**	**Percentage**	**Frequency**	**Percentage**	**Frequency**	**Percentage**	**Frequency**	**Percentage**	**Frequency**	**Percentage**			
*Q1*	0	0.0	0	0.0	2	33.3	3	50.0	1	16.7	3.8	0.8	0.059
*Q2*	0	0.0	1	16.7	2	33.3	2	33.3	1	16.7	3.5	1.0	.257
*Q3*	0	0.0	2	33.3	3	50.0	0	0.0	1	16.7	3.0	1.1	1.00
*Q4*	0	0.0	1	16.7	3	50.0	1	16.7	1	16.7	3.3	1.0	.414
*Q5*	0	0.0	0	0.0	0	0.0	3	50.0	3	50.0	4.5	0.5	.024
*Q6*	1	16.7	1	16.7	2	33.3	2	33.3	0	0.0	2.8	1.2	0.705
*Q7*	1	16.7	1	16.7	1	16.7	3	50.0	0	0.0	3.0	1.3	1.00
*Q8*	1	16.7	1	16.7	1	16.7	3	50.0	0	0.0	3.0	1.3	1.00
*Q9*	0	0.0	1	16.7	0	0.0	3	50.0	2	33.3	4.0	1.1	0.084
*Q10*	0	0.0	1	16.7	1	16.7	4	66.7	0	0.0	3.5	0.8	0.180
*Q11*	1	16.7	1	16.7	1	16.7	3	50.0	0	0.0	3.0	1.3	1.00
*Q12*	0	0.0	0	0.0	1	16.7	4	66.7	1	16.7	4.0	0.6	0.034
*Q13*	0	0.0	0	0.0	1	16.7	2	33.3	3	50.0	4.3	0.8	0.038
*Q14*	0	0.0	2	33.3	1	16.7	3	50.0	0	0.0	3.2	1.0	0.655
*Q15*	1	16.7	2	33.3	2	33.3	1	16.7	0	0.0	2.5	1.0	0.257
*Q16*	0	0.0	4	66.7	2	33.3	0	0.0	0	0.0	2.3	0.5	0.046
*Q17*	1	16.7	2	33.3	2	33.3	1	16.7	0	0.0	2.5	1.0	0.257
*Q18*	0	0.0	1	16.7	1	16.7	4	66.7	0	0.0	3.5	0.8	0.180
*Q19*	0	0.0	0	0.0	1	16.7	2	33.3	3	50.0	4.3	0.8	0.038
*Q20*	0	0.0	0	0.0	0	0.0	3	50.0	3	50.0	4.5	0.5	0.024
*Q21*	1	16.7	1	16.7	2	33.3	2	33.3	0	0.0	2.8	1.2	0.705
*Q22*	0	0.0	1	16.7	1	16.7	3	50.0	1	16.7	3.7	1.0	0.157
*Q23*	0	0.0	0	0.0	2	33.3	2	33.3	2	33.3	4.0	0.9	0.063
*Q24*	0	0.0	1	16.7	1	16.7	2	33.3	2	33.3	3.8	1.2	0.129
*Q25*	0	0.0	2	33.3	2	33.3	2	33.3	0	0.0	3.0	0.9	1.00
*Q26*	0	0.0	1	16.7	2	33.3	3	50.0	0	0.0	3.3	0.8	0.317
*Q27*	0	0.0	3	50.0	1	16.7	2	33.3	0	0.0	2.8	1.0	0.655
*Q28*	0	0.0	3	50.0	1	16.7	2	33.3	0	0.0	2.8	1.0	0.655
*Q29*	0	0.0	4	66.7	2	33.3	0	0.0	0	0.0	2.3	0.5	0.046
*Q30*	0	0.0	0	0.0	2	33.3	4	66.7	0	0.0	3.7	0.5	0.046

**TABLE 11:** Mean and median scores of teachers’ attitude.

	***N***	**Minimum**	**Maximum**	**Mean**	**Std. deviation**	**Median**	***p*-value**
Attitude	6	2.70	3.90	3.37	0.53	3.5	0.116

According to the results of the Table 10 and based on the Five Likert Scale, the highest mean score was seen in questions 5 and 20 (“Amuse learners” and “Being student-centered”) which was 4.5. On the other hand, the lowest mean score was found in questions 16 and 29 (“Waste one’s time” and “Increase anxiety”) which was 2.3. Thus, teachers believed that playing games would mostly affect two questions of the questionnaire, “Amuse learners” and “have student-centered classes.” However, teachers also stated that playing games does not play any role in two questions, “Increase anxiety,” and “Waste one’s time.”

The average score of questions in this section with the average of Likert Scale (3) was compared by using the Wilcoxon Test and it was revealed that the means of eight questions were significantly higher than 3 (*p* < 0.05). They were question 5 (“Amuse learners”), question 12 (“Help shy learners to participate”), question 13 (“Promote whole class participation”), question 16 (“Waste one’s time”), question 19 (“Enable learners to acquire new experiences”), question 20 (“Having student-centered classes”), question 29 (“Increase anxiety”), and question 30 (“Require little preparation”). In question number 20, the mean was significantly lower than 3. Therefore, the teachers’ answers for “Increase anxiety” was lower than average.

On the other hand, the means of the answers for seven questions were higher than the average. These questions were question 5 (“Amuse learners”), question 12 (“Help shy learners to participate”), question 13 (“Promote whole class participation”), question 16 (“Waste one’s time”), question 19 (“Enable learners to acquire new experiences”), question 29 (“Increase anxiety”), and question 30 (“Requires little preparation”). In the other questions, there was no significant difference in teachers’ answers with an average of 3, so teachers’ attitudes were about average in these questions.

Based on the Table 11 the teachers’ mean score for all the questions of the questionnaire was 3.37 and the Std. Deviation was 0.53. The Median was 3.5 which means that in half of the teachers, the score for the questionnaire was less than 3 and in the other half was more than 3. However, in comparing the median of the scores with the average of 3, no significant differences were seen (*p* > 0.05). Therefore, teachers’ attitude toward the treatment was moderate.

## Discussion

Although nowadays most teachers try to take advantage of new and encouraging methods in their L2 classrooms, it seems the implication of such methods has been overlooked in Iran. Therefore, an attempt has been made in this study to investigate the effect of playing games on Iranian learners’ willingness to communicate. To tackle the above problems the following research questions were addressed. One of the goals of this study is to answer the first research question: “does using games have a significant effect on Iranian EFL learners’ WTC?”

This question was followed by the hypothesis that using games does not have a significant effect on Iranian EFL learners’ WTC.

Considering the first research question, the results of the present study indicate that playing games in classes can have a significant effect on Iranian EFL learners’ willingness to communicate. According to Andrea (2011), games played a major role in achieving meaningful learning where the most productive and motivating learning experiences are taking place. This is in line with the findings of this study.

These findings reveal that games are very good at promoting students’ attitude towards learning English. They also reveal that by using games, learners learn the target language appropriately and enthusiastically. In addition, it increases their willingness to communicate when the topic of the lesson is interesting. Moreover, the results reveal that games are not just for fun, but also allow students to ask the teacher questions in class. These findings were in line with Modirkhameneh and Firouzmand (2014) as well as Lee and Drajati (2020). On the contrary, students believe that if they play games in classes, they do not dislike their classmates and they do not participate in pair activities in class. They also claim that they are not willing to talk when the teacher plays games.

In the present study, educational games are considered as important teaching tools that have not received enough attention in EFL classes. If the teachers play more games in classes, their students’ willingness to communicate will increase, which makes them more successful students. Therefore, the null hypothesis was rejected and using games has a significant effect on Iranian EFL learners’ WTC.

Another goal of the current study is to investigate the second research question: What are Iranian EFL teachers’ attitudes in regard to using games in increasing students’ WTC?

A unique finding of this study can be summed up in a short sentence “applying and choosing a game is a challenging task as it requires planning and effort as well as preparation; it amuses learners in students- centered classes.” This can be seen from the responses to items 5 and 20. Another unique finding is that games can help learners sustain interest and amuse all students in class. It received the highest level of importance. These findings were in line with Mahmoud and Tanni (2012).

Teachers believed that games amuse learners, help shy learners to participate, promote whole class participation, waste one’s time, enable learners to acquire new experiences, and have more student-centered classes. On the other hand, playing games does not increase anxiety; in this case the mean was significantly lower than 3 (*p* < 0.03). Therefore, the teachers’ answers for “Increase anxiety” were lower than average. Our findings were similar to Lindfors (1980), Ozmen (2004), and Millis (2005) who revealed that games can positively affect learning a foreign language. In contrast, this dimension receives the highest level of importance in the present study which might be considered as a unique finding. In terms of a communicative classroom where one feels comfortable, interested, and motivated, this study shares the assumption that games have a great effect in removing boredom. In this dimension, the present study is in line with other studies such as McFarlane and Sakellariou (2002); Fromme (2003) Thomas (2004); Cornelius-White (2007), Robbins and Timothy (2007); Prensky (2011), and Henry et al. (2021). Overall, the results have shown that a large number of participants believe that games can be used as an educational mechanism in English classrooms. There are certainly enough positive results to justify large-scale, extensive research into game playing habits, the motivations for playing games, and students’ attitude/perceptions towards games. In a nutshell, teachers believed that playing games increases Iranian learners’ willingness to communicate in the class and even out of the classroom, which is similar to de Freitas (2006) who claimed that games and simulations are very powerful and excellent tools that support collaborative learning skills.

## Conclusion

The findings of the present study demonstrate that games are effective as energizers and educational tools that can provide enjoyment, pleasure, passionate involvement, structure, and motivation among other benefits; the researchers supported the trend towards using them as short warm-ups.

When learning exercises are held alongside games, instruction is assisted, and increases foreign languages students’ achievement. Moreover, if English language is practiced with the help of games, the achievement of the learners can be higher than that from traditional education. This is a strong invitation for teachers to refer to games while teaching difficult tasks so as to maintain an interesting teaching environment.

Games should be perceived as elements of the process of teaching, learners should benefit from games connected with English learning in the process of teaching-learning at the right time and the right place.

The overall results of this study reflect the fact it does not matter what games are played; we cannot deny the importance of games. If students learn with games, have fun, and feel happy and free, it means that you have reached your goals. Games strengthen language skills, in addition to allowing learners to develop social skills and good relationships while they interact with each other.

Based on all of the information above it seems clear that games can and should be used as a teaching method when teaching languages. One reason why games could work well as a teaching method is because of the change that has occurred in teaching, where students have become much more active in the learning process. Besides giving students a chance to be more active, games usually place the teacher in a background role, and therefore allow the students to take on more responsibility.

When we consider the positive effects of language games, such as lowering learners’ anxiety and providing meaningful use of a language in the classroom, this result is striking and should be investigated in detail. Since the perspectives of learners and teachers might vary, even about the specific issue such as learning English through games, teachers and researchers should conduct studies or action research to examine learners’ views on several points to take into consideration when teaching a language and planning their lessons in a way that meets their individual learners’ needs. If learners are children, language teachers should not ignore their natural instincts for games, and they should seek ways to turn education into edutainment.

It has also been made clear that games help create diversity and can be very helpful in sustaining interest amongst students in school. We have also learned that by creating diversity, teachers are reaching out to a broader group of students and that is very important because students are individuals that differ from each other in so many ways.

## Data Availability Statement

The raw data supporting the conclusions of this article will be made available by the authors, without undue reservation.

## Ethics Statement

The studies involving human participants were reviewed and approved by the staff of Iranian Language Institutions. Written informed consent to participate in this study was provided by the participants.

## Author Contributions

All authors listed have made a substantial, direct and intellectual contribution to the work, and approved it for publication.

## Conflict of Interest

The authors declare that the research was conducted in the absence of any commercial or financial relationships that could be construed as a potential conflict of interest.

## Publisher’s Note

All claims expressed in this article are solely those of the authors and do not necessarily represent those of their affiliated organizations, or those of the publisher, the editors and the reviewers. Any product that may be evaluated in this article, or claim that may be made by its manufacturer, is not guaranteed or endorsed by the publisher.
